# Case Report: Bilateral vestibulopathy associated with leptomeningeal metastasis

**DOI:** 10.3389/fonc.2026.1981680

**Published:** 2026-10-20

**Authors:** Danyu Feng, Wan Wei, Shuqi Hu, Xiaoqin Hong

**Affiliations:** Department of Neurology, Affiliated Hangzhou First People’s Hospital, Westlake University School of Medicine, Hangzhou, China

**Keywords:** AQP4 antibody, bilateral vestibulopathy, leptomeningeal metastasis, lung adenocarcinoma, video head impulse test

## Abstract

**Introduction:**

Bilateral vestibulopathy (BVP) presents with oscillopsia, unsteadiness, and imbalance. Leptomeningeal metastasis (LM) is a rare and underrecognized cause of BVP, with direct infiltration of the vestibulocochlear nerves representing a potential mechanism of bilateral vestibular dysfunction. We report a case of LM-related BVP documented by quantitative video head impulse test (vHIT) of all six semicircular canals.

**Case report:**

A 61-year-old man presented with a six-month history of dizziness, tinnitus, oscillopsia, imbalance, and bilateral sensorineural hearing loss. vHIT showed reduced vestibulo-ocular reflex gain in all six semicircular canals. Contrast-enhanced MRI revealed leptomeningeal enhancement and enhancement of both vestibulocochlear nerves. CSF showed elevated protein, lymphocytic pleocytosis, and malignant cells. Serum anti-AQP4 antibodies were positive (1:320), but no other paraneoplastic antibodies were identified. He was diagnosed with LM from lung adenocarcinoma causing secondary BVP via direct infiltration of the vestibulocochlear nerves. Dizziness improved with intrathecal chemotherapy. However, he died approximately one year after discharge.

**Conclusion:**

BVP may be the initial presentation of LM. It underscores the necessity of considering rare etiologies in patients with unexplained BVP. Early recognition and targeted therapy may improve neurological outcomes, although overall prognosis remains limited in the setting of disseminated malignancy.

## Introduction

Bilateral vestibulopathy (BVP) involves hypofunction of the vestibular nerves or labyrinths on both sides, causing dizziness, oscillopsia, and unsteadiness, especially during movement and worsening in darkness or on uneven surfaces (1). BVP accounts for 4-7% of dizziness or vertigo, with a prevalence of 28 per 100,000 American adults (2). The main causes of BVP are ototoxic drugs, bilateral Menière’s disease, meningitis, and genetic disorders. The proportion of patients with a definite etiology varies among cohorts. Definite causes were identified in approximately 30% of a 255-patient cohort and 47% of a 154-patient cohort (1, 3).

BVP most commonly results from disorders affecting the peripheral vestibular system. Leptomeningeal metastasis (LM) is a rare but clinically important cause of BVP that may produce bilateral vestibular dysfunction through direct infiltration of the vestibulocochlear nerves. Its nonspecific clinical presentation overlaps with more common vestibular disorders and may therefore be mistaken for idiopathic BVP.

Here, we report a case of BVP secondary to LM from lung adenocarcinoma, accompanied by positive serum anti-AQP4-IgG, raising the question of a paraneoplastic versus a metastatic etiology. This case underscores the importance of considering neoplastic etiologies in patients presenting with unexplained bilateral vestibular dysfunction and highlights the need for comprehensive diagnostic evaluation, including cerebrospinal fluid (CSF) cytology and immunological testing, to guide timely and targeted management.

## Case report

A 61-year-old man was admitted to our neurology clinic with a six-month history of dizziness, recurrent tinnitus, oscillopsia, unsteadiness, and bilateral hearing loss. His dizziness and imbalance worsened on standing and in darkness, particularly at night. He had a past medical history of lung adenocarcinoma for more than 10 years and was treated with Osimertinib (160 mg QD). He reported no fever, otalgia, or ear discharge. There was no history of vestibulotoxic drug administration. The timeline diagram (**Figure 1**) illustrates the patient’s disease progression.

**Figure 1:**
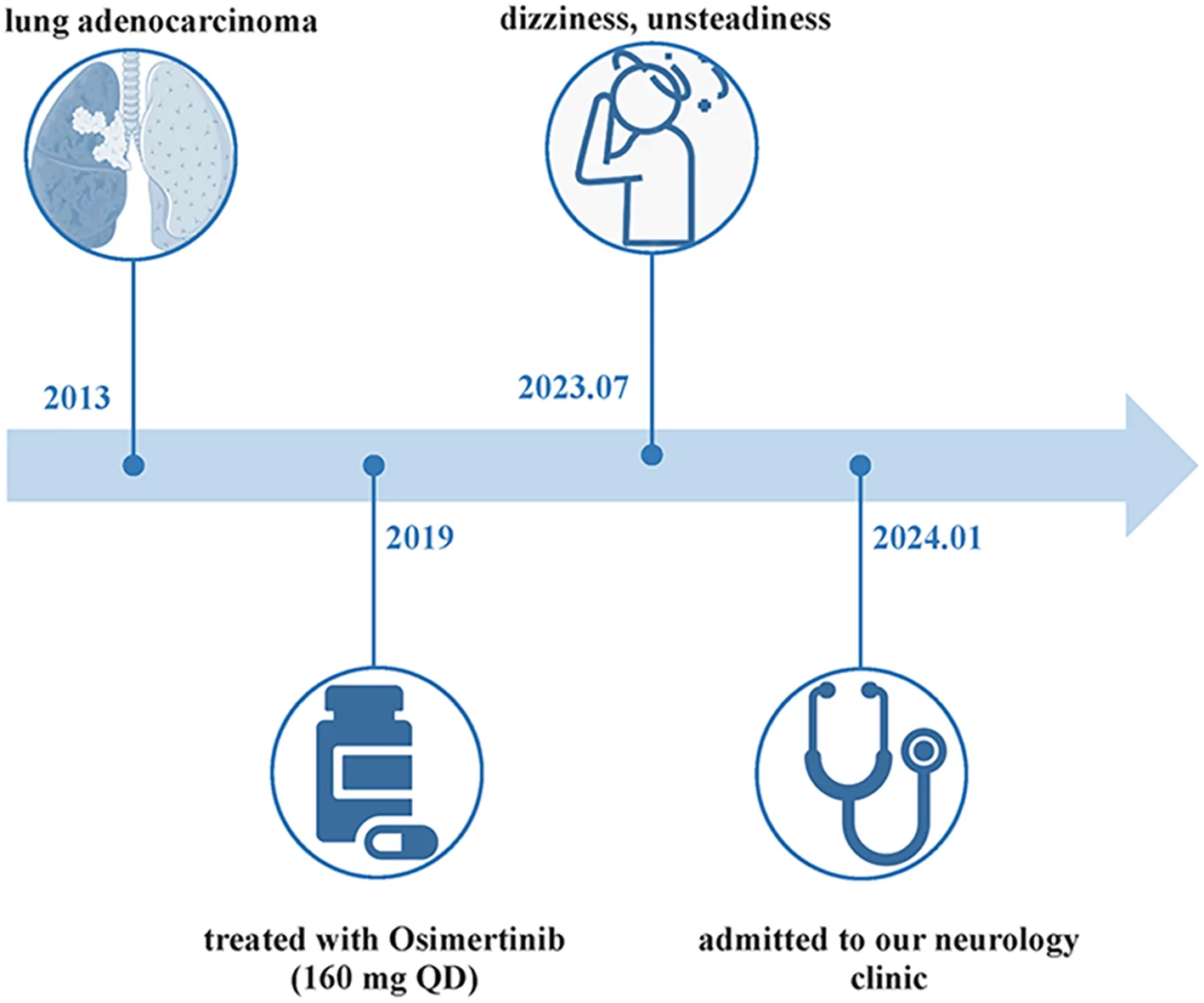
Clinical course. A timeline of disease progression.

The clinical examination showed a suspicious positive clinical horizontal head impulse test on the left, no spontaneous nystagmus, and reduced bilateral hearing to finger rub. Otoscopic examination showed patent external auditory canals and intact tympanic membranes bilaterally, with no discharge or signs of active infection. Both the Romberg and Tandem-Romberg signs were positive, but the Fukuda test could not be performed. All other neurological tests, including those for consciousness, cognition, speech, visual field deficits, pupillary response, facial palsy, muscle strength, sensation, ataxia, muscle tone, tendon reflexes, bilateral Babinski sign, roll test, Dix-Hallpike test, and meningeal irritation signs, were negative.

We conducted imaging and vestibular tests on the patient. The high-frequency video head impulse test (vHIT) confirmed vestibular impairment in all semicircular canals, with VOR gains below normal levels (**Figure 2C**). The audiogram (**Figures 2A, B**) showed bilateral sensorineural hearing loss, more severe on the right. Brainstem-evoked potentials were normal. A brain MRI with contrast showed bilateral vestibulocochlear nerve enhancement and leptomeningeal enhancement along the cerebral surfaces (**Figures 3A–E**). Spinal MRI detected metastases at T10, L1, and L4 (**Figure 3F**). Chest computed tomography (CT) on admission revealed a 22 × 13 mm mass-like high-density lesion in the anterior segment of the left upper lobe, which was considered suggestive of a tumor lesion (**Supplementary Figure 1**). CT and MRI plain scans confirmed normal cochlea and cochlear nerves (**Supplementary Figure 2**). An ear-nose-throat (ENT) consultation found no evidence of inner ear diseases, like Meniere’s syndrome, autoimmune inner ear disease, or labyrinthitis.

**Figure 2:**
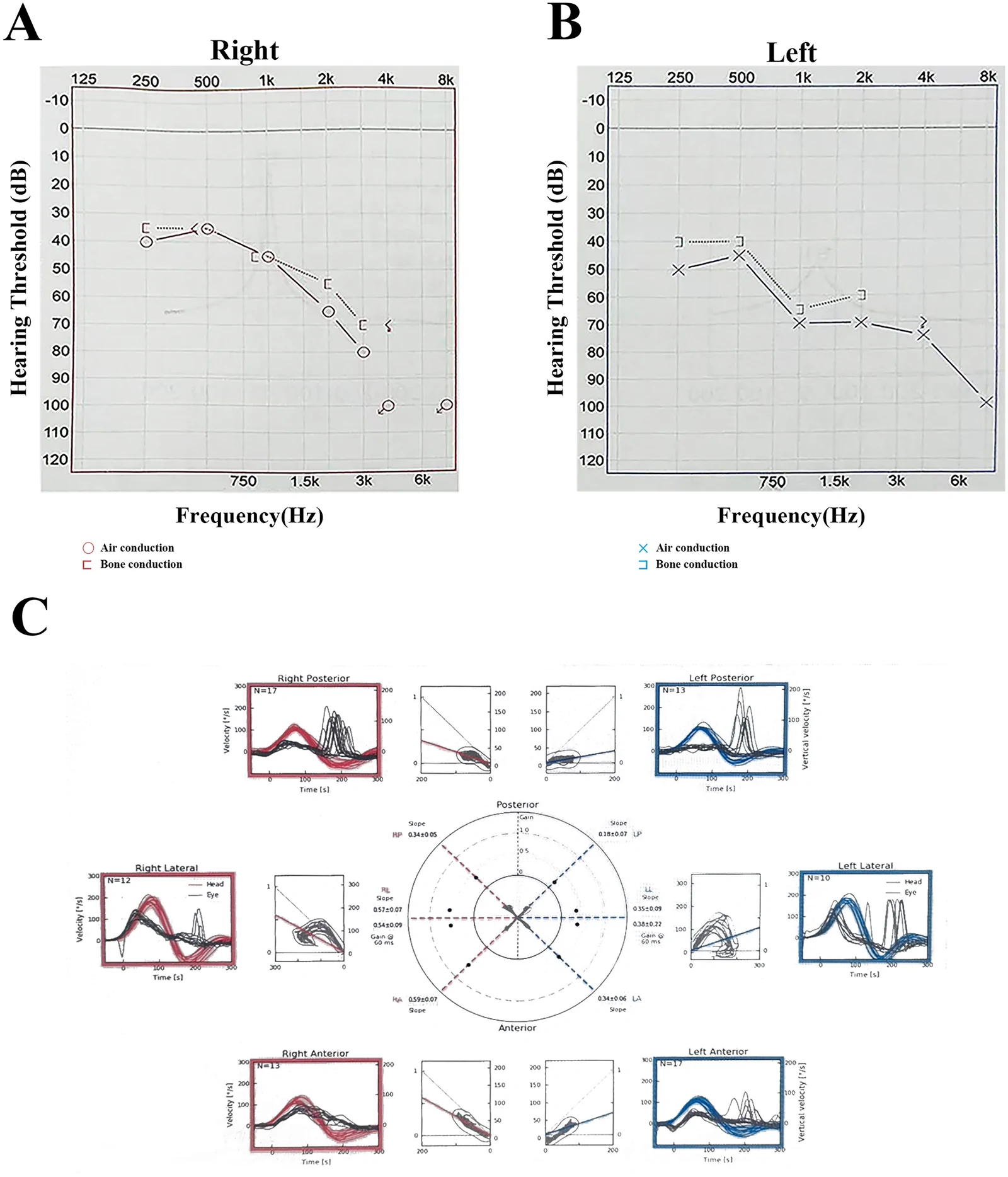
Audiometric and vestibular findings. **(A, B)** The audiogram confirmed bilateral sensorineural hearing loss. The right side **(A)** was more pronounced than the left **(B)**. **(C)** High-frequency video head impulse test (vHIT) confirmed vestibular impairment in the bilateral horizontal semicircular canals. The bilateral horizontal semicircular canals exhibited vestibulo-ocular reflex (VOR) gains of 0.54 ± 0.09 for the right and 0.38 ± 0.02 for the left, both below the normal threshold of >0.8. Similarly, the anterior semicircular canals demonstrated VOR gains of 0.59 ± 0.07 on the right and 0.34 ± 0.06 on the left, also below the normal range. The posterior semicircular canals showed VOR gains of 0.34 ± 0.05 for the right and 0.18 ± 0.07 for the left, which are likewise below the normal value of >0.8.

**Figure 3:**
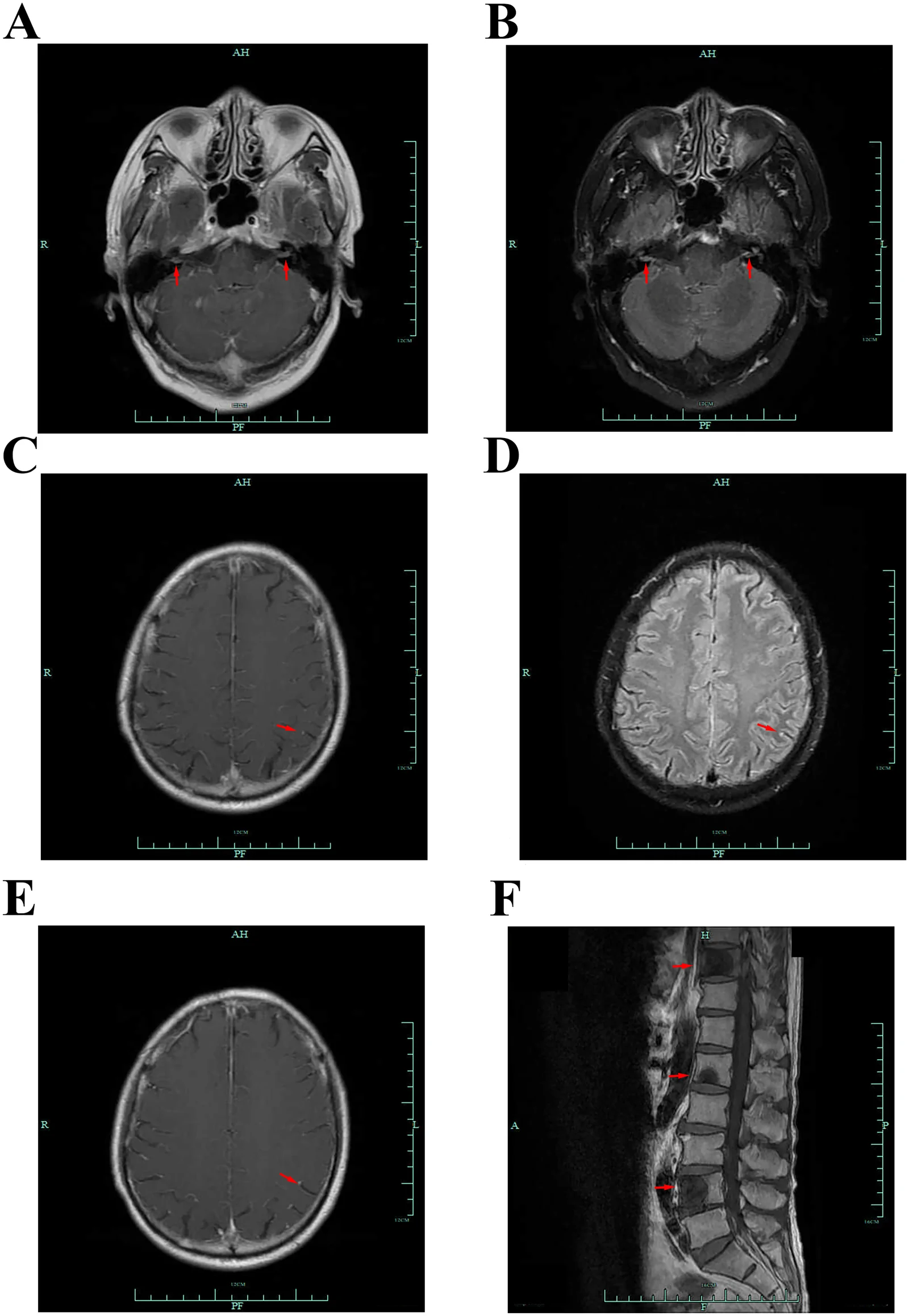
Brain and spinal MRI findings. **(A, C, E)** Axial T1-weighted postcontrast images. **(B, D)** Corresponding axial fat-suppressed T2-weighted postcontrast FLAIR images. **(A, B)** Bilateral enhancement of the vestibulocochlear nerves within the internal auditory canals is indicated by arrows. **(C–E)** Leptomeningeal enhancement along the cerebral surfaces is indicated by arrows. **(F)** Sagittal noncontrast T1-weighted thoracolumbar spinal MRI demonstrates vertebral metastases at T10, L1, and L4, as indicated by arrows.

To further clarify the diagnosis, we performed a lumbar puncture. The CSF showed normal pressure, glucose, and chloride levels, but the protein and immunoglobulin (IgG) were elevated at 110.2 mg/dL and 144 mg/L, respectively. The CSF contained 44 nucleated cells/μl (84% lymphocytes). The pathology report revealed cells with nuclear atypia and degeneration, individually. Tests for 30 paraneoplastic antibodies and autoimmune encephalitis are detailed in **Supplementary Tables S1**, **S2**. Notably, the serum anti-AQP4 antibody was positive with a titer of 1:320.

According to the diagnostic criteria for paraneoplastic neurologic syndromes, BVP in this patient is best explained by direct invasion of the vestibulocochlear nerves by LM rather than a paraneoplastic process. Under the PNS-Care scoring system, the patient received a score of 4, corresponding to possible paraneoplastic neurologic syndrome. Serum anti-AQP4-IgG contributed no points to the score. In addition, no other paraneoplastic antibodies were identified. Based on CSF cytology and contrast-enhanced brain MRI, the patient was ultimately diagnosed with LM from lung adenocarcinoma with secondary BVP.

He had vestibular function rehabilitation training. After being discharged, the patient sought follow-up care at another hospital’s neurosurgery department, where a lumbar puncture was carried out. Tumor cells were also detected in the cerebrospinal fluid after analysis. The therapeutic method was changed to a pemetrexed regimen, which consists of 15 mg of pemetrexed and 5 mg of dexamethasone given by intrathecal injection, when the initial Furmonertinib treatment proved insufficient. Following several therapy cycles, the patient’s dizzy symptoms subsided, and a subsequent study of the CSF revealed no discernible tumor cells. Since the patient received treatment in specialized departments at other hospitals, we could only track symptoms via phone follow-ups and case data from the family. Therefore, vHIT was performed only once. No follow-up brain or spinal MRI was performed, and no specific treatment was administered for the vertebral metastases. The patient subsequently declined further antitumor treatment and returned home. According to telephone follow-up with his family, he died approximately one year after discharge following clinical deterioration attributed to progressive malignancy.

## Discussion

In this case, the patient experienced bilateral vestibular dysfunction and hearing loss. Common causes of BVP include ototoxic medications, bilateral Ménière’s disease, and genetic disorders such as Deafness Autosomal Dominant (DFNA9). The patient had been receiving Osimertinib for lung cancer treatment since 2019. However, clinical trials of Osimertinib (4) reported no ototoxic side effects. The patient experienced dizziness and tinnitus in 2023, four years after starting the drug, which is much longer than typical ototoxic drug effects. Thus, targeted drugs are not considered the cause of the ototoxicity.

We tested 30 paraneoplastic antibodies, including KLHL11-IgG, linked to bilateral vestibular dysfunction, and amphiphysin-IgG (5). The high-risk antibodies KLHL11-IgG and ANNA1 (anti-Hu) were both negative. Under the PNS-Care scoring system (6), the patient received 4 points solely for the presence of cancer, meeting the numerical threshold for possible PNS. Serum anti-AQP4-IgG did not contribute points to the score because it is classified as a lower-risk antibody. Among patients with AQP4-IgG-positive NMOSD, cancer is reported in fewer than 5% of cases, with adenocarcinomas being the most commonly associated tumors (6). In a cohort study and literature review, patients with AQP4-IgG-positive NMOSD and temporally associated cancer were older at symptom onset and more frequently male than those without cancer (7). Although our patient was a 61-year-old man with lung adenocarcinoma, he had no core clinical characteristics of NMOSD, such as optic neuritis, acute myelitis, area postrema syndrome, or characteristic brainstem, diencephalic, or cerebral syndromes (8, 9). Isolated bilateral vestibular dysfunction is not a typical manifestation of AQP4-IgG-associated disease. Moreover, the PNS-Care criteria require reasonable exclusion of alternative etiologies before a neurologic syndrome is attributed to a paraneoplastic mechanism. Accordingly, serum AQP4-IgG positivity was considered an accompanying serological finding. It did not establish NMOSD or support a paraneoplastic cause of the bilateral vestibular and auditory impairment.

Paraneoplastic cochleovestibulopathy and LM cannot be reliably distinguished by vestibulocochlear nerve enhancement alone. Internal auditory canal enhancement was reported in 4 of 26 patients (15%) with paraneoplastic cochleovestibulopathy (5). Conversely, LM may initially present with isolated bilateral enhancement of vestibulocochlear nerves. Diffuse leptomeningeal involvement may become apparent only on subsequent imaging, as reported by Santarelli and Di Paola (10). In a cohort of 114 patients with meningeal carcinomatosis, contrast-enhanced 3D-BRAVO imaging detected involvement of the combined facial and auditory nerve group in 58.77% of patients. Trigeminal nerve involvement was detected in 47.37%, whereas optic nerve involvement occurred in 5.26% (11). These findings indicate that accompanying abnormalities and their evolution are more informative than cranial nerve enhancement alone. Therefore, in our patient, the combination of leptomeningeal enhancement, bilateral vestibulocochlear nerve enhancement, and malignant cells in the CSF favored LM over paraneoplastic cochleovestibulopathy.

LM is an uncommon cause of bilateral vestibular impairment (1). Our literature review identified six previously reported cases in which bilateral vestibular dysfunction was objectively documented, all of which were individual case reports (12–17). These cases are summarized and compared with the present case in **Table 1**. An additional 23 publications described auditory or vestibular symptoms associated with LM but did not provide objective evidence of bilateral vestibular hypofunction. These reports are summarized separately in **Supplementary Table S3**. Among the six previously reported cases, bilateral vestibular dysfunction was demonstrated by caloric testing in three cases, rotary harmonic stimulation combined with electronystagmography in one, electronystagmography combined with dynamic posturography in one, and caloric testing followed by a bedside head impulse test in one. In our patient, vestibular loss was quantitatively assessed using vHIT, which demonstrated reduced vestibulo-ocular reflex gains in all six semicircular canals. Comprehensive data concerning the fundamental epidemiological characteristics of LM remain insufficient. However, limited institutional reviews and autopsy studies suggest that the incidence rate of LM in advanced malignant tumors ranges from approximately 9% to 25% for both small cell and non-small cell lung cancer (18). Metastatic cells in cerebrospinal fluid can spread along the meninges or fluid flow, preferring areas with slow flow or gravity dependence, and sites of fluid absorption or discharge. The vestibular nerve, exiting the brainstem at the cerebellopontine angle, passes through slow-flow areas, facilitating tumor colonization. Wrapped in pia mater, the vestibular and auditory nerves traverse these regions, making them susceptible to meningeal transfer and CSF-flow, leading to vestibular nerve damage (18). The pathophysiology most consistent with this presentation is direct metastatic seeding of the vestibulocochlear nerves via the CSF rather than a remote paraneoplastic effect. This hypothesis is further corroborated by our enhanced MRI findings (**Figure 3**).

**Table 1:** Published cases of leptomeningeal metastasis with objectively documented bilateral vestibular dysfunction.

No.	Author (year)	Primary tumor	Age/Sex	Vestibular manifestations	Auditory manifestations	Evidence of bilateral vestibular dysfunction	Neuroimaging	CSF	Treatment	Outcome
1	Shioya et al. (1990) (12)	Gastric cancer	74/M	Wide-based ataxic gait. Jumbling phenomenon.	Progressive bilateral deafness	Bithermal caloric testing showed absent responses bilaterally.	MRI NR. Cranial CT was unremarkable.	CEA was elevated. Malignant cells were detected 4 months after admission.	NR	Progressive complete bilateral loss of auditory and vestibular function. Survival NR.
2	Houck et al. (1992) (13)	Breast carcinoma	65/F	Mild unsteadiness. No vertigo, nausea, or vomiting at onset.	Sudden profound bilateral SNHL with loud buzzing	Rotary harmonic stimulation and ENG showed absent vestibular function bilaterally.	Initial head CT and MRI were normal, including the temporal bones. Follow-up CT showed periventricular and left parietal metastases.	Malignant cells resembled those in the breast carcinoma specimen. Protein was greater than 200 mg/dL.	Ommaya reservoir. Palliative chemotherapy and radiotherapy.	Died approximately 4 months after the sudden hearing loss.
3	Shen et al. (2000) (14)	Lung adenocarcinoma	57/M	Dizziness followed by ataxic gait	Progressive bilateral high-frequency SNHL with tinnitus	Initial vestibular testing and ENG were normal. Repeat ice-water caloric testing showed a weak response on the right and no response on the left.	Leptomeningeal enhancement involved the posterior fossa and bilateral temporal lobes. The left CN VIII enhanced.	Adenocarcinoma cells were detected.	Whole-brain irradiation	Died 1 year after the diagnosis of leptomeningeal metastasis.
4	Pollak et al. (2001) (15)	Breast carcinoma	73/F	Progressive gait unsteadiness	Bilateral tinnitus	ENG and dynamic posturography supported the authors’ diagnosis of bilateral vestibular failure.	Bilateral enhancement of CN VIII	Malignant cells were detected.	Intrathecal methotrexate	No response at 10 weeks. Died 22 weeks after treatment began.
5	Wagemakers et al. (2005) (16)	Esophageal signet-ring cell carcinoma	52/M	Disequilibrium and head-movement-dependent oscillopsia	Profound bilateral hearing loss	Bithermal caloric testing showed bilateral vestibular areflexia.	Bilateral CN VIII and right CN V swelling, T2 hyperintensity, and enhancement. Leptomeningeal enhancement appeared later.	Cytology did not confirm malignancy.	Skull-base radiotherapy	Symptoms progressed. Died 16 weeks after the onset of hearing loss.
6	Kim et al. (2015) (17)	Lung adenocarcinoma	49/F	Persistent vertigo followed by worsening oscillopsia	Rapidly progressive bilateral SNHL with tinnitus	Initial caloric testing showed 75% right canal paresis. Later bedside HIT showed bilateral catch-up saccades, indicating bilateral vestibulopathy.	Irregular nodular enhancement in both IACs with leptomeningeal enhancement and multiple small parenchymal metastases	Initial cytology was negative. Later cytology was suspicious for malignant cells. CSF CEA was elevated.	Gefitinib and WBRT at 30 Gy in 10 fractions	Tumor showed a partial response. Hearing and vestibular dysfunction progressed.
Present case	Current report	Lung adenocarcinoma	61/M	Dizziness, oscillopsia, and unsteadiness for 6 months	Bilateral SNHL with recurrent tinnitus	vHIT showed reduced VOR gain in all six semicircular canals.	Leptomeningeal and bilateral CN VIII enhancement. Spinal metastases at T10, L1, and L4.	Malignant cells were detected. No discernible tumor cells were seen after treatment.	Furmonertinib followed by intrathecal pemetrexed and dexamethasone	Dizziness improved. Died approximately 1 year after discharge following progressive malignancy.

Inclusion criterion. A report was included only when bilateral vestibular hypofunction was documented by caloric testing, rotary testing, ENG, HIT, or vHIT.

BAER, brainstem auditory evoked response. CEA, carcinoembryonic antigen. CN, cranial nerve. CSF, cerebrospinal fluid. CT, computed tomography. ENG, electronystagmography. HIT, head impulse test. IAC, internal auditory canal. MRI, magnetic resonance imaging. NR, not reported. RT, radiotherapy. SNHL, sensorineural hearing loss. vHIT, video head impulse test. VOR, vestibulo-ocular reflex. WBRT, whole-brain radiotherapy.

In this case, the patient was identified with LM secondary to lung cancer using cerebrospinal fluid analysis, and bilateral vestibular dysfunction with hearing loss was a sign of cranial nerve involvement. During follow-up, the patient received intrathecal administration of pemetrexed. The symptoms of vestibular dysfunction improved in conjunction with negative cerebrospinal fluid results. Nonetheless, owing to the reduced survival probability linked to meningeal metastases (19), the patient died following disease progression over one year post-diagnosis.

## Conclusion

We describe a case of bilateral vestibulopathy associated with LM from lung adenocarcinoma with AQP4-IgG positivity. It is crucial to meticulously identify the underlying cause of BVP, since addressing this disease may result in both subjective and objective improvements. LM should be considered in patients with progressive dizziness and unsteadiness due to otherwise unexplained BVP, particularly when there is a history of malignancy. Contrast-enhanced MRI and CSF cytology are important for identifying the underlying cause.

## Data Availability

The raw data supporting the conclusions of this article will be made available by the authors, without undue reservation.
