# Supplementary material for: Case Report: Bilateral vestibulopathy associated with leptomeningeal metastasis
Source: Front Oncol. 2026 Oct 20;16:1981680. doi: 10.3389/fonc.2026.1981680 (PMC13727439; doi:10.3389/fonc.2026.1981680)
Supplement: Supplementary file 1 [file Table2.docx]

**Supplementary Table S3. Reports of leptomeningeal metastasis with auditory or vestibular symptoms but without objectively documented bilateral vestibular dysfunction**

| **No.** | **Author (year)** | **Primary tumor** | **Age/Sex** | **Vestibular manifestations** | **Auditory manifestations** | **Vestibular function testing** | **Neuroimaging** | **CSF or pathology** | **Treatment** | **Outcome** |
| --- | --- | --- | --- | --- | --- | --- | --- | --- | --- | --- |
| 1 | Civantos et al. (1992) (1) | Poorly differentiated esophageal squamous cell carcinoma | 68/M | NR | Complete sudden bilateral hearing loss | NR | NR | Numerous poorly differentiated malignant cells | Intrathecal methotrexate and cisplatin. Skull-base radiotherapy. | Died on hospital day 25. |
| 2 | Uppal et al. (2001) (2) | Occult breast carcinoma | 61/F | NR | Sudden profound bilateral SNHL | NR | NR | Malignant cells detected | Dexamethasone and tamoxifen | Died 11 weeks after presentation. |
| 3 | Testoni et al. (2005) (3) | Melanoma | 53/M | No vestibular symptoms reported | Isolated symmetric progressive bilateral hearing loss | NR | Dural thickening, diffuse subarachnoid enhancement, nodular IAC lesions, and a right parietal lesion | Inflammatory cells and S-100-positive malignant cells | NR | NR |
| 4 | Suzuki et al. (2006) (4) | Rectal adenocarcinoma | 60/F | NR | Progressive left SNHL followed by rapid profound right SNHL | NR | Left IAC and CPA tumor with right IAC enhancement. Rapid right-sided tumor growth was later seen. | Malignant cells consistent with poorly differentiated adenocarcinoma | NR | Died approximately 4 months after diagnosis. |
| 5 | Jeffs et al. (2006) (5) | Metastatic melanoma | 66/F | NR | Sudden bilateral SNHL | NR | Bilateral thickening and enhancement of CN VIII within the IACs | NR | Steroids and urgent radiotherapy | Hearing did not improve. Died 2 weeks after hearing-loss onset. |
| 6 | Astner et al. (2007), two cases (6) | Unknown primary in one case. Esophageal cancer in one case. | 60/M and 70/M | Vertigo reported | Rapid bilateral hearing loss | NR | Bilateral IAC or CPA lesions with additional cranial-nerve lesions in one case | Atypical cells reported | Craniospinal radiotherapy in one case. Intrathecal methotrexate and radiotherapy in one case. | One patient died during therapy. The other had partial remission for several months and died 6 months after hearing-loss onset. |
| 7 | Okamoto et al. (2007) (7) | Poorly differentiated gastric adenocarcinoma | 63/M | NR | Rapidly progressive bilateral deafness | NR | Bilateral hypertrophic and enhancing CN VII and CN VIII roots | Atypical cells reported. Diagnosis confirmed at autopsy. | NR | Died on day 12 after admission. |
| 8 | Koda et al. (2008) (8) | Squamous cell carcinoma of the lower esophagus | 63/M | NR | Bilateral sudden deafness | NR | Tumors involving both CN VIII nerves and the right CN V | NR | NR | Died of pneumonia 5 months after the first symptom. |
| 9 | Goyault et al. (2009), prospective cohort (9) | Mixed solid tumors | 14 patients. 7 F and 7 M. Age 20 to 84 years. | Individual vestibular symptoms NR | SNHL in 5 of 14 patients | No individual vestibular function data reported | IAC enhancement in 14 of 14. Inner-ear enhancement in 3 of 14, all with SNHL. Two additional patients with isolated IAC enhancement had SNHL. | NR | NR | NR |
| 10 | Mourgela et al. (2009) (10) | Small-cell lung cancer | 56/M | NR | Progressive bilateral SNHL with left facial palsy | NR | Bilateral internal acoustic meatus lesions with skull-base leptomeningeal enhancement | Cytology positive for malignant cells | Whole-brain radiotherapy and intrathecal methotrexate | NR |
| 11 | Vitaliani et al. (2009) (11) | Ovarian carcinoma | 63/F | Vertigo, walking imbalance, positive Romberg sign, and bilateral gaze-evoked nystagmus | Right deafness with tinnitus followed by left deafness | NR | Cerebellar pial enhancement and bilateral CN VII and CN VIII enhancement with a right nodular IAC lesion | Neoplastic cells detected | Supportive care | Died 20 days after admission. |
| 12 | Ohno et al. (2010) (12) | Gastric cancer | 62/M | Mild dizziness | Sudden bilateral SNHL | NR | Numerous metastatic nodules with bilateral CN VIII thickening and enhancement within the IACs | NR | Oral S-1, weekly paclitaxel, and radiotherapy | Died 12 weeks after the onset of deafness. |
| 13 | Marchese et al. (2010), two cases (13) | Occult malignancy in both cases | 56/M and 64/F | Vertigo reported | Sudden or progressive SNHL | NR | Bilateral IAC and CPA masses. One case also had dural thickening, diffuse subarachnoid enhancement, and a right parietal lesion. | Atypical cells reported in both cases. Histopathology or surgical biopsy established metastatic disease. | Systemic corticosteroids in both cases. Chemotherapy and stereotactic radiotherapy in one case. | Rapid progression. One patient died within days of diagnosis. Complete follow-up for the other case was NR. |
| 14 | Blows et al. (2012) (14) | Pancreatic adenocarcinoma | 72/M | Bilateral vestibulocochlear nerve palsies were described, but specific vestibular symptoms were NR | Sudden bilateral deafness | NR | Brain MRI was unremarkable apart from mild small-vessel ischemic change | Reactive macrophages without malignant cells. Leptomeningeal infiltration was confirmed post mortem. | Intravenous dexamethasone | Died 3 weeks after admission. |
| 15 | Kim et al. (2014) (15) | Gastric signet-ring cell carcinoma | 58/M | Dizziness, vertigo, and vomiting | Bilateral hearing difficulty | NR | Multiple enhancing lesions along the cisternal and canalicular segments of both CN VIII nerves | CSF cytology negative. Diagnosis established by surgical biopsy. | Radiotherapy at 3000 cGy in 10 fractions | Died from pneumonia 1 week after surgery. |
| 16 | Öztürk et al. (2014) (16) | Duodenal signet-ring cell adenocarcinoma | 44/M | Dizziness and diplopia | Sudden bilateral SNHL | NR | Bilateral CN VIII enhancement | Malignant cells detected | Cranial radiotherapy | Died 3 months after hospitalization. |
| 17 | de Mones del Pujol et al. (2014), six-case series (17) | Lung adenocarcinoma in two cases, breast adenocarcinoma, melanoma, ethmoid adenocarcinoma, and unknown primary | Six patients | Vestibular symptoms were the first complaint in five cases | Rapid hearing decline and bilateralization in five cases | No individual objective bilateral vestibular test results reported | Bilateral IAM lesions in five cases and a unilateral IAM lesion in one case | Malignant CSF cytology in three cases, tumor biopsy in one case, and imaging-supported diagnosis in two cases | Whole-brain radiotherapy in five cases and intrathecal methotrexate in one case | Rapidly fatal in five cases. One patient remained disease free for 4 years after treatment. |
| 18 | Krupa and Byun (2017) (18) | Ovarian carcinoma | 62/F | No specific vestibular symptoms reported | Bilateral sensorineural deafness with right facial weakness | NR | Bilateral IAC masses and leptomeningeal enhancement | Initial cytology negative. Repeat CSF cytology was positive for metastatic adenocarcinoma. | Chemotherapy | NR |
| 19 | Kimura et al. (2019) (19) | Poorly differentiated gastric adenocarcinoma | 66/M | Slight dizziness and right-beating nystagmus followed by vertigo | Left profound hearing loss followed by right hearing loss and bilateral deafness | NR | Bilateral gadolinium-enhancing CPA and IAC masses | Adenocarcinoma cells detected | Prednisolone was given initially. No leptomeningeal-directed treatment was possible after rapid deterioration. | Died 46 days after hearing-loss onset. |
| 20 | Tanaka et al. (2020) (20) | Gastric signet-ring cell carcinoma | 68/M | Dizziness | Bilateral deafness and tinnitus | NR | Tumor filled both IACs | Class V adenocarcinoma cells detected | No leptomeningeal-directed treatment | Died approximately 2 months after symptom onset. |
| 21 | Alsalem et al. (2022) (21) | Breast adenocarcinoma | 72/F | No focal vestibular symptoms reported | Rapidly progressive bilateral SNHL | NR | Bilateral IAC enhancement and left cochlear enhancement | Undifferentiated carcinoma cells and Zic4 antibodies detected | Intrathecal methotrexate | No improvement in hearing. |
| 22 | Agarwal et al. (2024) (22) | Metastatic gastric cancer | Late 40s/M | Dizziness and vomiting | Acute severe bilateral SNHL | NR | Bilateral thickening and peripheral enhancement of the intracanalicular facial and vestibulocochlear nerve complexes | Highly atypical epithelial cells detected | Intrathecal methotrexate | Died from sudden cardiac arrest 2 weeks after the first chemotherapy dose. |
| 23 | Lin et al. (2026) (23) | Gastric cardia adenocarcinoma | 71/M | Dizziness and unsteady gait | Rapidly progressive bilateral hearing loss | NR | Extensive cerebral and cerebellar leptomeningeal enhancement with bilateral CN VIII enhancement | Cytology negative. Markedly reduced glucose and elevated protein. | Best supportive care | Rapid deterioration to impaired consciousness. Survival NR. |

**Abbreviations.** BVP, bilateral vestibulopathy. CN, cranial nerve. CPA, cerebellopontine angle. CSF, cerebrospinal fluid. ENG, electronystagmography. HIT, head impulse test. IAC, internal auditory canal. IAM, internal auditory meatus. MRI, magnetic resonance imaging. NR, not reported. SNHL, sensorineural hearing loss. vHIT, video head impulse test.

**Supplementary References**

1.	Civantos F, Choi YS, Applebaum EL. Meningeal carcinomatosis producing bilateral sudden hearing loss: A case report. *Vis Inf Commun Interact* (1992) 13:369–71.

2.	Uppal HS, Ayshford CA, Wilson F. Sudden onset bilateral sensorineural hearing loss: A manifestation of occult breast carcinoma. *J Laryngol Otol* (2001) 115:907–910. doi: 10.1258/0022215011909323

3.	Testoni S, Pirodda A, Pastore Trossello M, Minguzzi E, D’Alessandro R. Meningeal carcinomatosis causing isolated bilateral symmetric progressive hearing loss. *Neurol Sci* (2005) 25:345–347. doi: 10.1007/s10072-004-0369-2

4.	Suzuki T, Sakaguchi H, Yamamoto S, Hisa Y. Sudden hearing loss due to meningeal carcinomatosis from rectal carcinoma. *Auris Nasus Larynx* (2006) 33:315–319. doi: 10.1016/j.anl.2005.11.010

5.	Jeffs GJ, Lee GY-F, Wong GT-H. Leptomeningeal carcinomatosis: An unusual cause of sudden onset bilateral sensorineural hearing loss. *J Clin Neurosci* (2006) 13:116–118. doi: 10.1016/j.jocn.2004.12.009

6.	Astner ST, Nieder C, Stock K, Gaa J, Grosu AL. Carcinomatous meningitis appearing as acoustic neuromas: Two cases. *Strahlenther Onkol* (2007) 183:279–283. doi: 10.1007/s00066-007-1615-3

7.	Okamoto Y, Sugiyama H, Yamamoto T. [a case of meningeal carcinomatosis presenting with bilateral hearing loss]. *Vis Inf Commun Interact* (2007) 59:1385–9.

8.	Koda H, Kimura Y, Iino Y, Eishi Y, Murakami Y, Kitamura K. Bilateral sudden deafness caused by diffuse metastatic leptomeningeal carcinomatosis. *Otol Neurotol* (2008) 29:727–729. doi: 10.1097/mao.0b013e3181758eb1

9.	Goyault G, Kremer S, Martin E, Riehm S, Dietemann J-L, Veillon F. Leptomeningeal carcinomatosis and sensorineural hearing loss: correlation of labyrinthine enhancement patterns with symptoms. *J Neuroradiol* (2009) 36:98–101. doi: 10.1016/j.neurad.2008.11.003

10.	Mourgela S, Sakellaropoulos A, Ardavanis A. Leptomeningeal carcinomatosis presenting as bilateral sensorineural deafness and unilateral facial palsy. *J Buon* (2009) 14:317–9.

11.	Vitaliani R, Spinazzi M, Del Mistro AR, Manara R, Tavolato B, Bonifati DM. Subacute onset of deafness and vertigo in a patient with leptomeningeal metastasis from ovarian cancer. *Neurol Sci* (2009) 30:65–67. doi: 10.1007/s10072-008-0006-6

12.	Ohno T, Yokoyama Y, Aihara R, Mochiki E, Asao T, Kuwano H. Sudden bilateral sensorineural hearing loss as the presenting symptom of meningeal carcinomatosis of gastric cancer: Report of a case. *Surg Today* (2010) 40:561–565. doi: 10.1007/s00595-009-4099-1

13.	Marchese MR, La Greca C, Conti G, Paludetti G. Sudden onset sensorineural hearing loss caused by meningeal carcinomatosis secondary to occult malignancy: Report of two cases. *Auris Nasus Larynx* (2010) 37:515–518. doi: 10.1016/j.anl.2009.11.003

14.	Blows SJ, Morgan R, Dhariwal U, Petts G, Roncaroli F. Pancreatic adenocarcinoma presenting with sudden onset bilateral deafness secondary to metastatic leptomeningeal infiltration. *Age Ageing* (2012) 41:818–819. doi: 10.1093/ageing/afs085

15.	Kim S-J, Kwon J-T, Mun S-K, Hong Y-H. Leptomeningeal carcinomatosis of gastric cancer misdiagnosed as vestibular schwannoma. *J Korean Neurosurg Soc* (2014) 56:51. doi: 10.3340/jkns.2014.56.1.51

16.	Öztürk M, Ila K, Düzgöl C, Akansel G, Almaç A. Bilateral sudden sensorineural hearing loss caused by leptomeningeal carcinomatosis: Case report and review. *Vis Inf Commun Interact* (2014) 24:287–291. doi: 10.5606/kbbihtisas.2014.37029

17.	de Mones del Pujol E, Kouassi J, Maire J-P, Franco-Vidal V, Darrouzet V. Meningeal carcinomatosis of the internal auditory meatus: clinical and imagery-aided differentiation. *Otol Neurotol* (2014) 35:911–917. doi: 10.1097/mao.0000000000000308

18.	Krupa M, Byun K. Leptomeningeal carcinomatosis and bilateral internal auditory canal metastases from ovarian carcinoma. *Vis Inf Commun Interact* (2017) 12:386–390. doi: 10.1016/j.radcr.2017.03.013

19.	Kimura A, Takahashi Y, Mizutari K, Tsujimoto H, Nakanishi K, Shiotani A. A case of gastric meningeal carcinomatosis involving bilateral hearing loss: the difference between clinical images and autopsy findings. *J Int Adv Otol* (2019) 15:333–336. doi: 10.5152/iao.2019.6080

20.	Tanaka T, Kanetaka K, Ikeda T, Yamaguchi S, Kawakami S, Kitajima T, Iwata T, Eguchi S. A rare case of meningeal carcinomatosis and internal auditory canal metastasis presenting with the deafness for gastric cancer. *Vis Inf Commun Interact* (2020) 6:295. doi: 10.1186/s40792-020-01018-1

21.	Alsalem A, Marzoughi S, Chen T. Clinical Reasoning: A 72-Year-Old Woman With Rapidly Progressive Bilateral Hearing Loss. *Neurology* (2022) 98:632–637. doi: 10.1212/wnl.0000000000200009

22.	Agarwal A, Garg D, Priya S, Bylappa PS, Garg A, Shamim SA, Elhence A, Radhakrishnan DM, Pandit AK, Srivastava AK. Acute bilateral sensorineural hearing loss as presentation of leptomeningeal metastases. *Pract Neurol* (2024) 24:407–409. doi: 10.1136/pn-2024-004167

23.	Lin Y, Li X, Zhan X, Dong W. Case report: extensive leptomeningeal metastasis from gastric cancer presenting with hearing impairment. *Front Oncol* (2026) 16:1888134. doi: 10.3389/fonc.2026.1888134
